# A Case Report of Aortic Dissection Involving the Aortic Root, Left Common Carotid Artery, and Iliac Arteries

**DOI:** 10.21980/J8V93K

**Published:** 2022-01-15

**Authors:** Miguel Angel Martinez-Romo, Christopher Eric McCoy

**Affiliations:** * University of California, Irvine, Department of Emergency Medicine, Orange, CA

## Abstract

Acute aortic dissection is a life-threatening event caused by separation of the aortic layers that requires prompt management and surgical consultation. We present the case of a 53-year-old male who developed acute, severe chest pain radiating to his back at a community hospital and was transferred to a tertiary center for definitive surgical management. The patient’s aortic dissection was diagnosed via computed tomography angiography. He was started on rate-control and blood pressure medications, and was admitted emergently to the operating room. Emergency physicians should obtain immediate surgical consultation, promptly start medications for rate and blood pressure control, and administer analgesia in order to stabilize their patient and decrease the shear forces that would further propagate an aortic dissection.

**Topics:**

Aortic dissection, cardiothoracic surgery, vascular surgery, hypertensive emergency, aorta.

[Fig f1-jetem-7-1-v13] [Fig f2-jetem-7-1-v13] [Fig f3-jetem-7-1-v13] [Fig f4-jetem-7-1-v13] [Fig f5-jetem-7-1-v13] [Fig f6-jetem-7-1-v13] [Fig f7-jetem-7-1-v13]

**Figure f1-jetem-7-1-v13:**
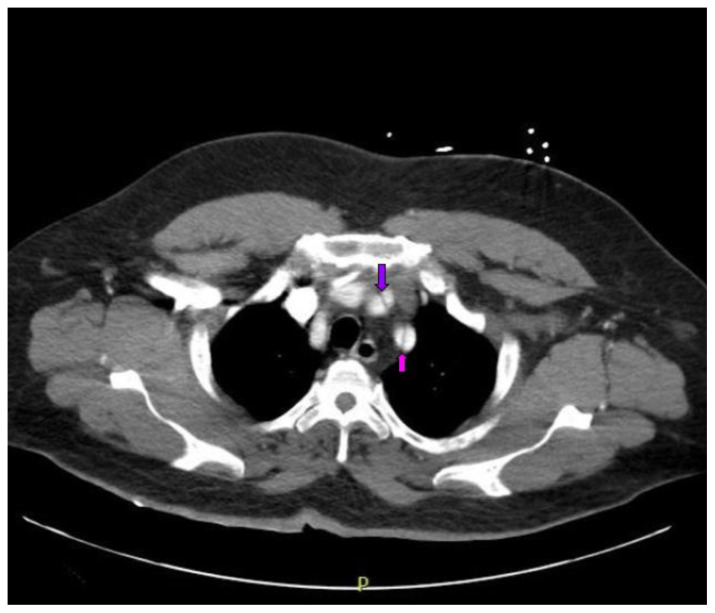


**Figure f2-jetem-7-1-v13:**
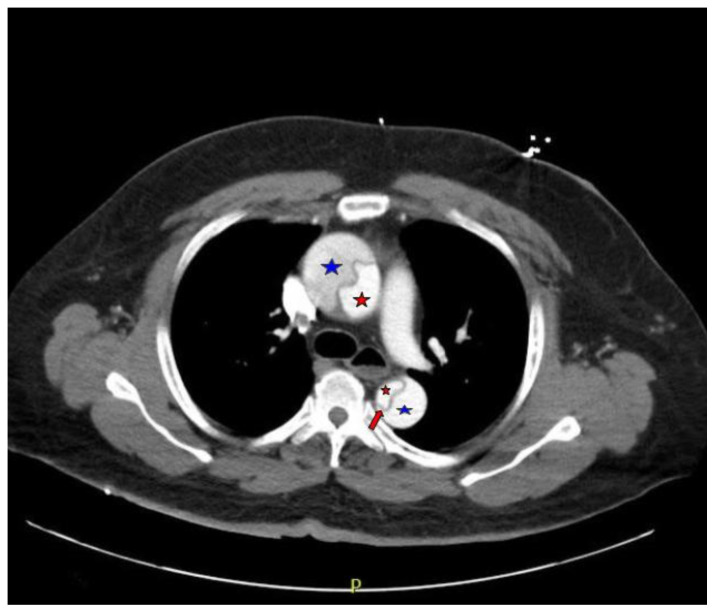


**Figure f3-jetem-7-1-v13:**
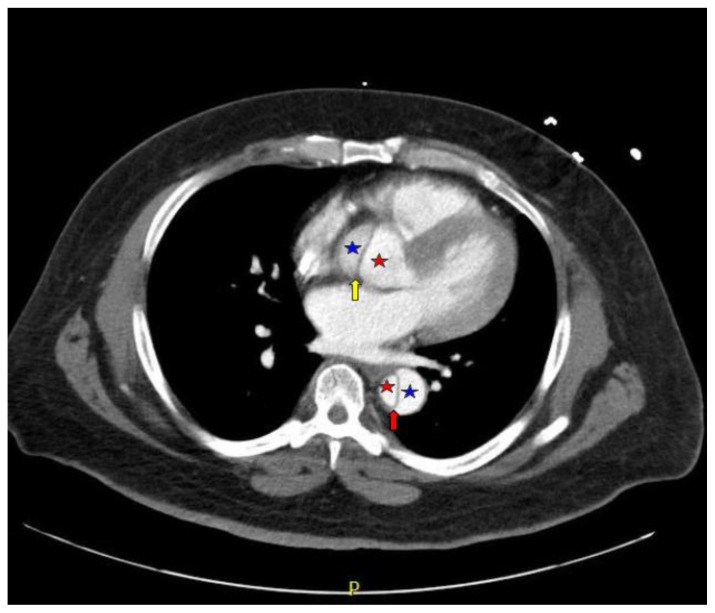


**Figure f4-jetem-7-1-v13:**
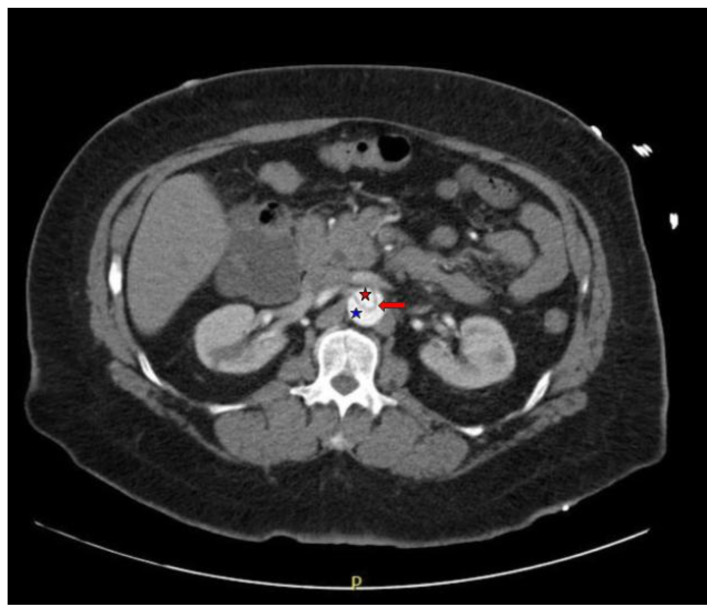


**Figure f5-jetem-7-1-v13:**
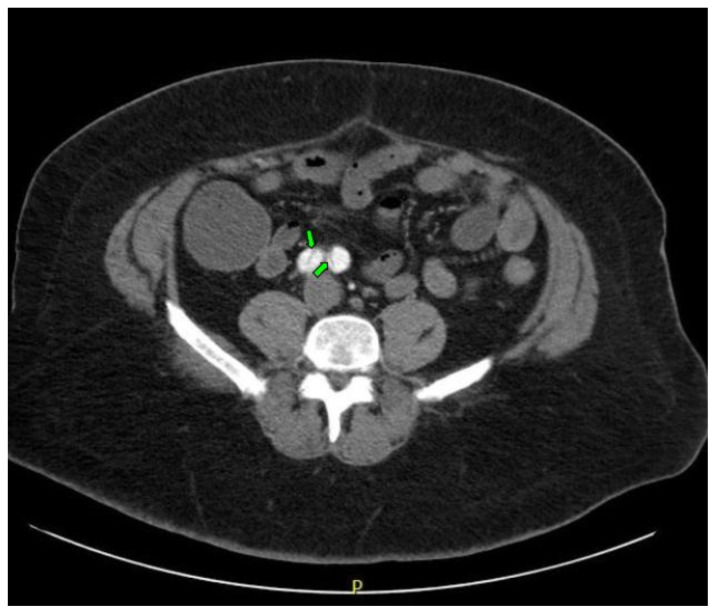


**Figure f6-jetem-7-1-v13:**
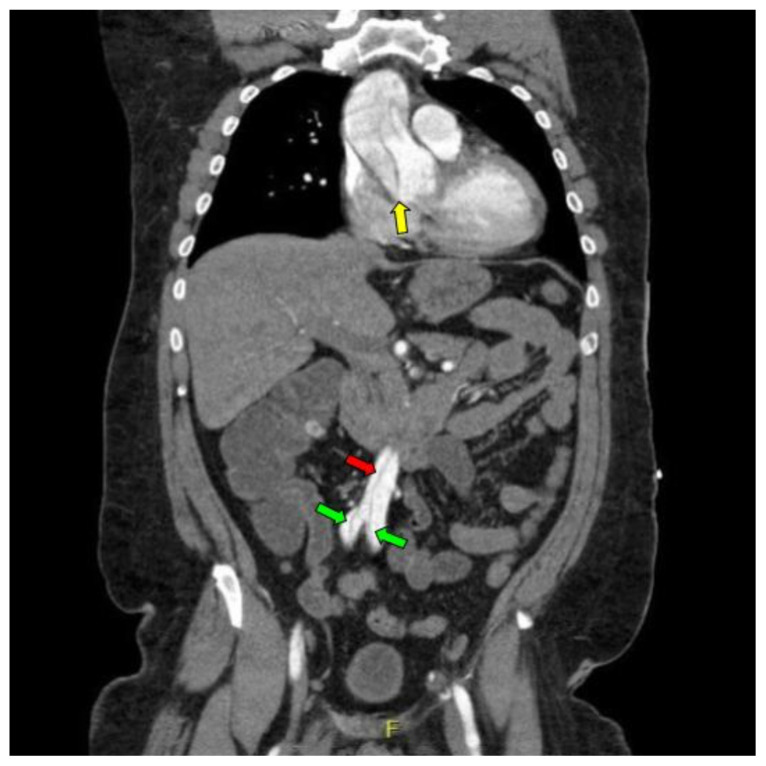


**Figure f7-jetem-7-1-v13:**
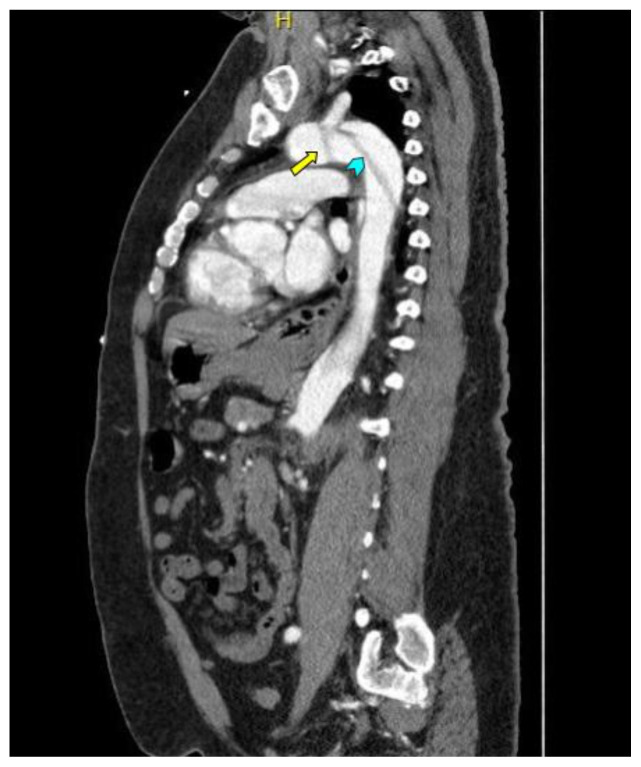


## Brief introduction

Acute aortic dissection (AoD) is separation of the layers of the aorta.^1^ The aorta is separated into 3 layers, the intima (innermost layer), media (middle), and adventitia (outermost).^1^ In an aortic dissection, the media layer of the aorta is disrupted resulting in subsequent blood dissecting through the plane within the media.^1^ ‘Acute aortic dissection occurs within less than 2 weeks of the onset of symptoms, while subacute dissection occurs within 2 to 6 weeks of symptom onset, and chronic dissection occurs more than 6 weeks after onset of symptoms.^1^ Acute aortic dissection (AoD) is most commonly due to intimal disruption that dissects through a plane within the media. Other causes include aortic intramural hematomas (IMH) and penetrating atherosclerotic ulcers (PAU).^1^ This case report will present an illustrative case of a patient suffering an extensive aortic dissection of the ascending and descending aorta and introduce the management options available for this condition.

## Presenting concerns and clinical findings

A 53-year-old male, with a past medical history of hypertension and cerebrovascular accident (CVA) without any residual deficits, was transferred to a tertiary center from a community hospital after developing severe substernal chest pain radiating to his back for one day. He was diagnosed with an aortic dissection on computed tomography angiography (CTA), started on a nitroprusside drip by the outside community hospital, and transferred to the tertiary center’s emergency department (ED) after coordination with cardiothoracic surgery. The tertiary hospital’s patient care coordinator notified the Operating Room (OR) prior to the patient’s arrival and the OR was prepared for him before he even arrived. However, the cardiothoracic team wanted the patient to stop in the ED first to optimize medical management prior to the OR given the patient’s high blood pressure. Upon presentation to the tertiary center, the patient’s initial vitals were a temperature (T) 36.4 **°**C, a blood pressure (BP) of 210/120 mmHg, a heart rate (HR) of 70 beats/min, a respiratory rate (RR) of 16/minute, and an oxygen saturation (O_2_ Sat) of 95% on room air. The patient was immediately placed in the ED’s resuscitation room and placed on a cardiac monitor with pulse oximetry. Exam revealed a middle-aged man in moderate distress due to pain, normal cardiac rate with no murmurs, equal peripheral pulses, and no neurological deficits. The ED physicians started the patient on esmolol for rate control and added a nicardipine drip to achieve a blood pressure goal of 100–120 mmHg systolic. He received opioid analgesia with intravenous morphine and the emergency resident performed a bedside ultrasound which revealed no pericardiac effusion. The patient had a brief ED stay once rate and blood pressure control were achieved since the OR was already prepared for him prior to his arrival. He was admitted to the operating room by cardiothoracic surgery for emergent aortic repair.

## Significant findings

Computed tomography angiography (CTA) of the thoracic and abdominal aorta revealed an aortic dissection of the ascending aorta, with a dissection flap starting from the aortic root/aortic annulus (yellow arrows), extending into the aortic arch (light blue arrowhead) and involving the left common carotid artery (purple arrow), left subclavian artery (pink arrow), extending to the descending aorta (red arrows), and into the bilateral iliacs (green arrows). The true lumen (red star) and false lumen (blue star) created by the dissection flap can best be seen in the axial views. Laboratory findings revealed a mild leukocytosis with a white blood cell (WBC) count of 14.6 (reference range 4.0–10.5 WBC’s × 10^9^ per L), but were overall insignificant with a lactate level of 1.3 mmol/L (0.5–2.0 mmol/L) and a high sensitivity troponin I of 13 ng/L (0–20 ng/L).

## Patient course

Patient had an aortic root replacement and an ascending aorta repair with a tube-graft performed by cardiothoracic surgery. Vascular surgery was consulted for the dissection involving the descending aorta, and they recommended medical management. His surgery was complicated by postoperative respiratory insufficiency in which he failed three spontaneous breathing trials, becoming apneic and hemodynamically labile. He also had postoperative acute blood loss anemia, acute kidney injury, coagulopathy, thrombocytopenia, and postoperative hypoglycemia. On post-operative day 1 he was finally extubated, and his inpatient stay was further complicated by intermittent delirium, for which he was started on olanzapine, and episodes of atrial fibrillation, for which he was started on metoprolol and bridged to warfarin. After a lengthy hospitalization, the patient’s mental status improved and he was neurologically intact. However, the patient was severely debilitated, only able to move out-of-bed with the help of the Physical Therapist. The patient was discharged to a skilled-nursing facility given his extensive rehabilitation and medical management requirements.

## Discussion

The incidence of AoD is 2–3.5 cases per 100,000 person-years, which correlates to 6,000 to 10,000 cases annually in the United States.^1^ AoD has a high mortality-rate, with approximately 40% dying immediately, 1% dying per hour thereafter without intervention, and 5–20% dying during or after surgery.^1^ Particular bad outcomes are found in the elderly.^2^

The clinical presentation of AoD is variable. However, abrupt, severe chest and/or back pain is the most common presentation, followed by abdominal pain with or without chest or back pain. Other presentations include painful or numb lower extremities, neurological deficits, and altered-level of consciousness.^1^,^3^ Although classically described as occurring in hypertensive patients, the International Registry of Acute Aortic Dissection (IRAD) database revealed that 45% of patients were normotensive and 14% were hypotensive.^1^,^3^ Those with hypertension, connective tissue disorders (Marfan’s, Ehlers-Danlos syndrome), genetic mutations that cause thoracic aneurysms, family history of dissection or aneurysm, aortic valve disease, known thoracic aneurysms, and recent aortic manipulation are at highest risk.^1^ Diagnosis in the emergency department is usually done through Computed Tomography Angiography (CTA) of the aorta,^2^ which also assists in categorizing the dissection according to the DeBakey or Stanford classification.^1^ Laboratory tests have no role in the diagnosis AoD. 1 D-dimers have been used in some studies to aid in the diagnosis of AoD; however, the guidelines of thoracic aortic disease state that D-dimers cannot be used to “rule out” AoD. 1 Management is surgical and/or medical, and is dependent on the DeBakey or Stanford classification, patient clinical status, or symptoms.^1^ AoD involving the ascending aorta should be urgently evaluated for surgical repair.^1^ AoD involving the descending aorta (without involving the ascending aorta) should be managed medically, unless complications develop.^1^ Complications include aortic insufficiency, cardiac tamponade, heart failure, ischemic stroke, spinal ischemia, mesenteric ischemia, limb ischemia, and death.^1^ However, urgent surgical consultation should be made for AoD as soon as the diagnosis is made or strongly suspected, regardless of anatomic location (ascending or descending).^1^ Medical management involves initiating intravenous beta-blockers to achieve target heart-rate of 60 beats-per-minute or less; esmolol is a good first-line option.^1^ If systolic blood-pressure remains greater than 120 mm Hg after beta-blockade from rate control, then a vasodilator and/or an angiotensin-converting enzyme inhibitor should be used intravenously to achieve target systolic blood-pressure between 100–120 mmHg.^1^ Vasodilators should not be initiated prior to rate-control to avoid reflex tachycardia that may increase aortic wall stress.^1^ It is also important to decrease sympathetic surge through intravenous opiate analgesia for pain control; pain can increase both heart-rate and blood pressure.^1^

## Supplementary Information














