# Supplementary figures and images for: A Case Report of Aortic Dissection Involving the Aortic Root, Left Common Carotid Artery, and Iliac Arteries
Source: J Educ Teach Emerg Med. 2022 Jan 15;7(1):V13–7. doi: 10.21980/J8V93K (PMC10358868; doi:10.21980/J8V93K)

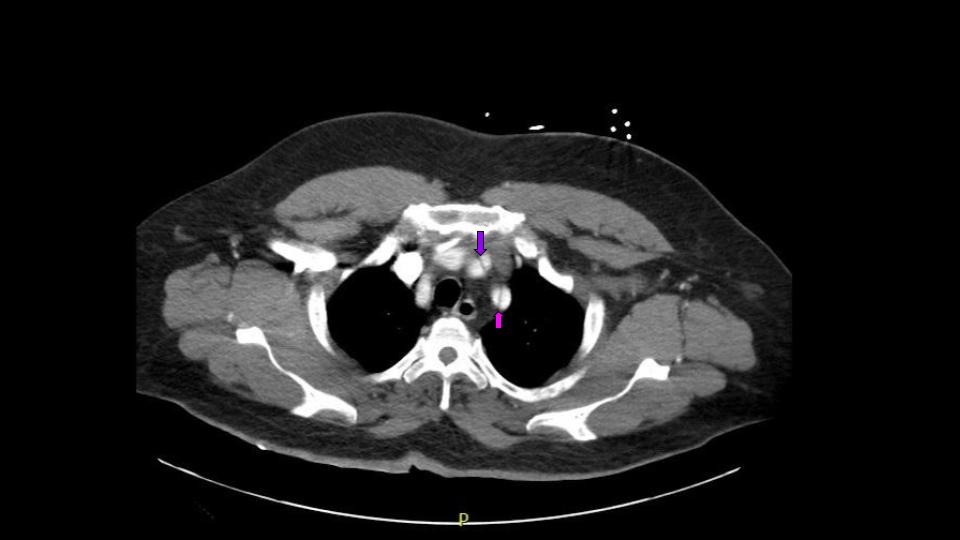

Supplement: Supplementary file 1 [file JETem-7-1-V13-supp1.jpg]

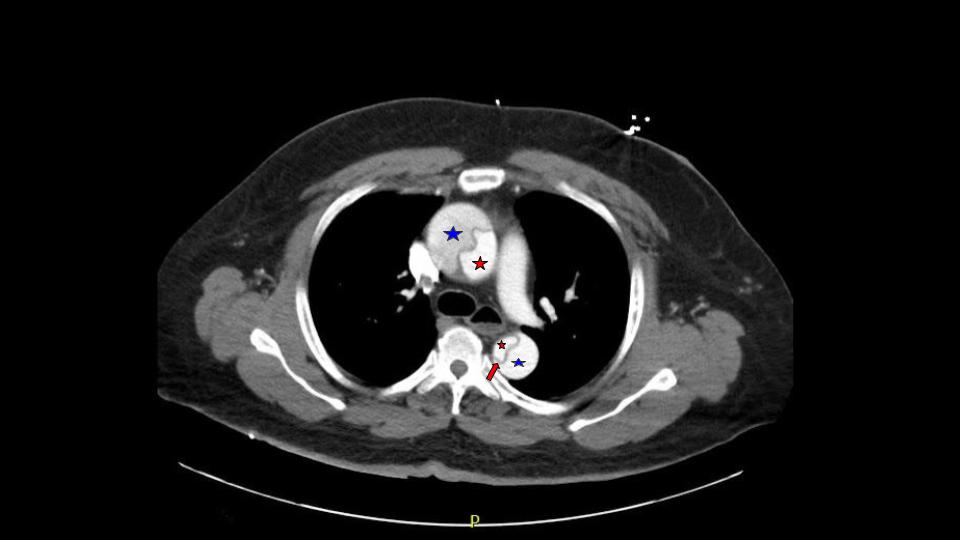

Supplement: Supplementary file 2 [file JETem-7-1-V13-supp2.jpg]

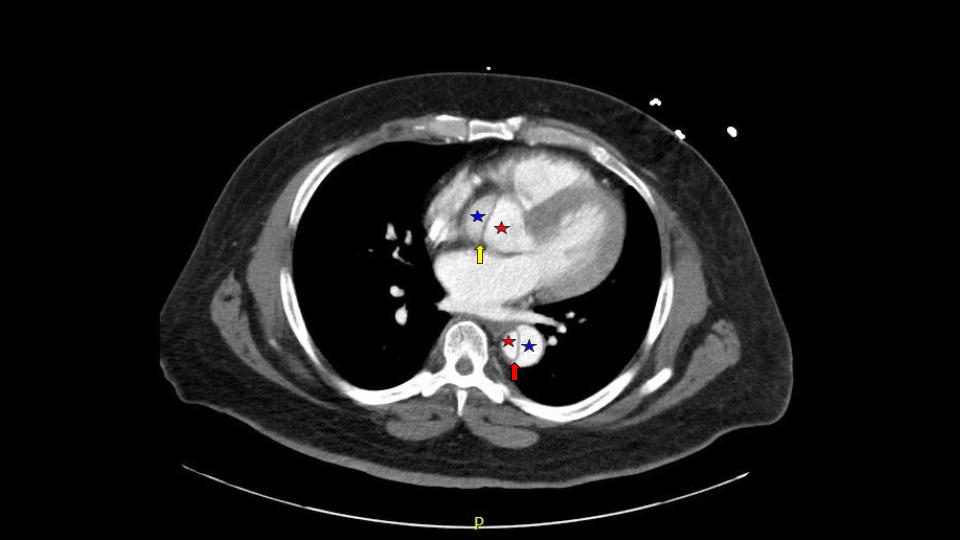

Supplement: Supplementary file 3 [file JETem-7-1-V13-supp3.jpg]

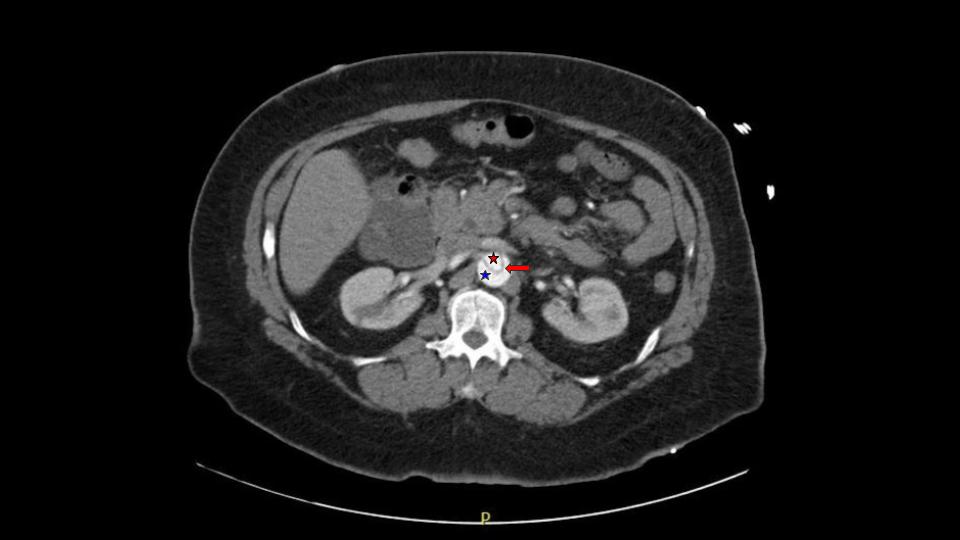

Supplement: Supplementary file 4 [file JETem-7-1-V13-supp4.jpg]

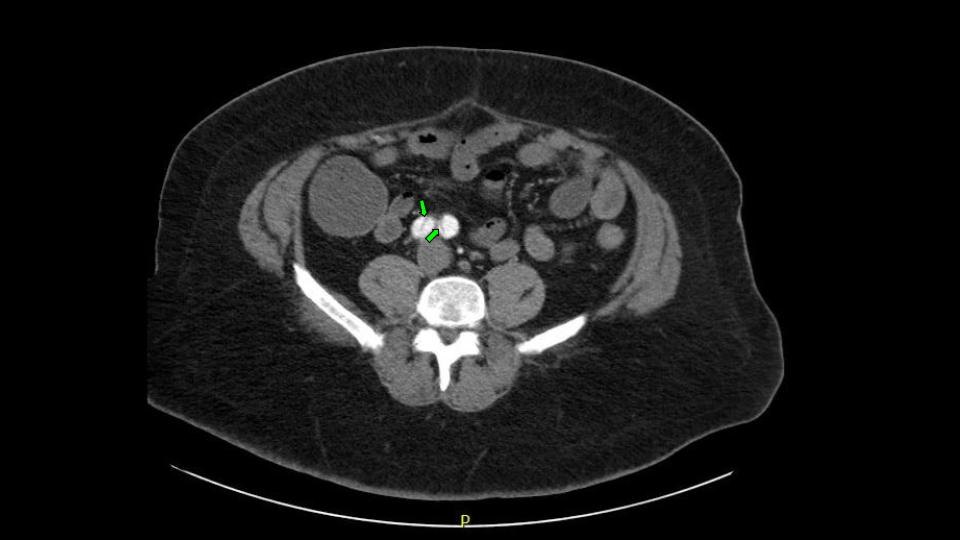

Supplement: Supplementary file 5 [file JETem-7-1-V13-supp5.jpg]

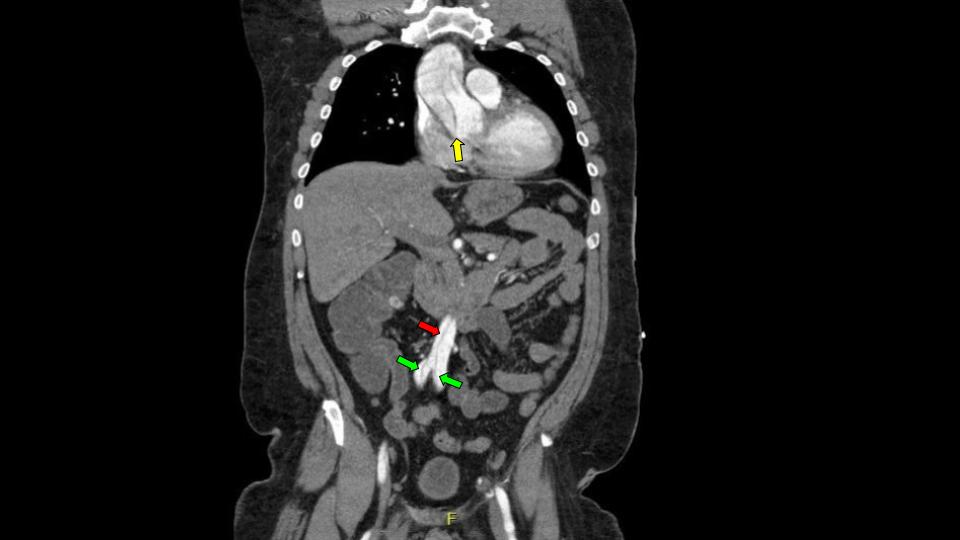

Supplement: Supplementary file 6 [file JETem-7-1-V13-supp6.jpg]

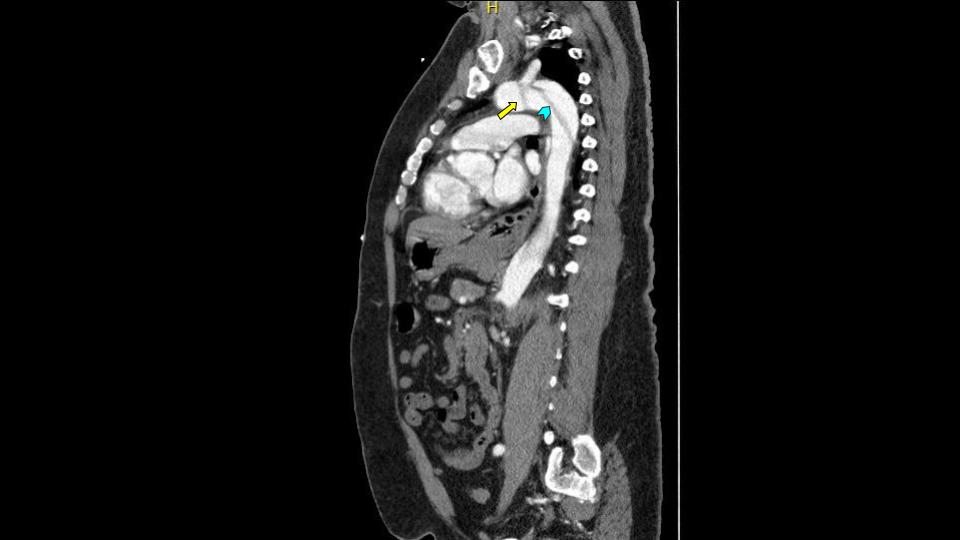

Supplement: Supplementary file 7 [file JETem-7-1-V13-supp7.jpg]
